# Hong Kong’s new wave of migration: socio-political factors of individuals’ intention to emigrate

**DOI:** 10.1186/s40878-022-00323-y

**Published:** 2022-12-04

**Authors:** Anita Kit Wa Chan, Lewis T. O. Cheung, Eric King-man Chong, Man Yee Karen Lee, Mathew Y. H. Wong

**Affiliations:** 1 The Department of Social Sciences, The Education University of Hong Kong, Lo Ping Road, Tai Po, Hong Kong; 2 La Trobe Law School, La Trobe University, Melbourne, VIC 3086 Australia

**Keywords:** Migration, Migration intention, Mobility, Sense of place, Global citizenship, Inequality, Trust and confidence in the law and the legal system

## Abstract

With a recent surge in the outward movement of the population, a new wave of emigration has been suggested to have started in Hong Kong. It is speculated that recent socio-political changes in Hong Kong may have contributed to this phenomenon. Therefore, five socio-political variables—mobility, sense of place, trust and confidence in the law and the legal system, global citizenship, and perception of inequality—are employed in this study as proposed determinants to investigate the intention of Hong Kong residents to migrate to mainland China and to other international destinations. A random telephone questionnaire survey representative of the local population was conducted, with a total of 801 valid samples collected. Stepwise multiple regression analysis was carried out. The results showed that all five proposed socio-political variables successfully predicted people’s migration intention to mainland China and to foreign countries, with important variations between the two choices. Our results carry strong implications for understanding people’s concerns behind their intention to emigrate. Further, our findings present a challenge for Hong Kong; society may gradually be failing to accommodate individuals with diverse perceptions and values, particularly in terms of trust and confidence in the law and the legal system, and individuals’ sense of global citizenship.

## Introduction

In 1997, Hong Kong was officially handed back to China by Britain and the Hong Kong Special Administrative Region (HKSAR) was established. Prior to 1997, increasing emigration rates were documented (Census and Statistics Department n.d.), marking this period as featuring one of the most recent waves of emigration in Hong Kong, with great societal changes accompanying it. In 2019, a series of social movements concerning the proposed Anti-Extradition Law Amendment Bill, which would allow criminal suspects to be sent from Hong Kong to stand trial in mainland China, broke out in Hong Kong. They were met by unyielding government responses and, ultimately, the enactment and implementation of the Law of the People’s Republic of China on Safeguarding National Security in the Hong Kong Special Administrative Region (the National Security Law) in July 2020. This series of ongoing events has brought with it drastic changes and instability in Hong Kong, to no lesser extent than those that occurred during the previous waves of emigration. At the same time, countries such as the UK, Canada, and Australia have started to offer new immigration channels to Hong Kong residents, with lower requirements and restrictions. According to the UK’s Home Office, there were up to 88,000 successful applications for British National (Oversea) (BNO) visas in the period from January 2021 to September 2021, as new immigration policies were announced by the UK government (GOV.UK, 2021). Amid the COVID-19 pandemic, with highly restricted border crossings, a historical peak of net outward movement of populations was still seen in Hong Kong in 2020 and 2021. The total net movement of the population was made up by − 85,000 and − 75,000 individuals in 2020 and 2021, respectively, which is particularly remarkable given that these figures have rarely involved negative numbers in the past 10 years (Census and Statistics Department n.d.). A survey conducted by the Hong Kong Institute of Asia–Pacific Studies (2021) also showed that, the proportion of Hong Kong citizens with intentions to emigrate rose from 33% in 2017 to 42% in 2021. For these reasons, both the public and the media have started to question if a new wave of migration has emerged.

Despite the escalating outward migration rate in recent years, Hong Kong, as a world-class international city, has been and is likely still a desirable destination for immigration. Population figures also show that the net inward movement of the population after 1997 has mostly been positive (Census and Statistics Department n.d.). With immigration from mainland China dominating the movements of the population, migration studies in Hong Kong have primarily focused on immigration and Chinese immigrants (Chan & Ngan, 2018; Lee & Liang, 2020; Ngan & Chan, 2021), while investigations of emigration and relevant contributing factors are relatively scarce. As one of the few exceptions, a recent study by Lui et al. (2022) investigates the relationships between emigration, politics and family decisions under the current macro-political conditions and climate of Hong Kong. As migration is facilitated based on the dynamics between the migration origin and the migration destination (De Haas, 2011), it is crucial to understand the factors making Hong Kong a migration origin, especially under the recent trend of emigration. In fact, studies on migration worldwide have also suffered from failing to pay sufficient attention to the origins of migration; De Haas (2011) argues that existing theories and approaches in studying migration generally lack considerations regarding factors that reflect the influence of the origin of migration (e.g., on market structure, income inequality, conflict, and policies, etc.). This study therefore aims to identify the potential determinants of the migration origin in promoting migration, which may shed light on the local situation, as well as fill the current gap in existing research.

The current trend of migration in Hong Kong can hardly be defined by and explained in terms of typical legal and official categories of migration or migrants (e.g., labor, economic, or refugee migration and migrants), as the issue is highly contextual and individual motives likely play a more significant role than typically well-studied determinants such as economic factors. In terms of the approaches taken in studies of migration, De Haas (2011) has suggested that traditional approaches, such as neo-classical migration or the new economics of labor migration (NELM), are generally macro- and economic-oriented, and fail to take into account social and contextual factors, as well as individual motivations. Instead, De Haas (2011) has recommended studying people’s motivations to migrate from a socio-psychological perspective, which would acknowledge people as active agents of migration and take into account factors from structural to individual levels. Through this approach, a behavioral link between micro- and macro-level factors, that is generally lacking in macro-level studies can be formed to address the behavioral assumption of such studies. In other words, determinants at macro levels, like demographic changes or environmental degradation, could be correlated to migration, yet these determinants do not actually explain the behavior or motivation of migration (De Haas, 2011). For instance, a place with extreme rich-poor gap may have higher rate of migration. Yet, the concept or indicators of rich-poor gap in a macro sense does not explain or lead people to migrate. Instead, the perceptions and experiences of people with inequality, e.g., the perceived lack of opportunities, that could be inquired through a socio-psychological perspective, would motivate people to migrate. Notably, it is the aggregation of migration behaviors caused by such perceptions and motivations of individuals that would produce the outcomes or the measurements and indicators at a macro level. Some existing literature have also taken on a non-economic perspective in studying migration and have discussed the influence of various determinants and motivations of migration, such as education (Sandu et al., 2018), family (Thomas, 2019), self-exploration (Ono, 2009), social security (Sana & Massey, 2000), and political stability and systems (Aziz et al., 2021; Etling et al., 2020; Khosa & Kalitanyi, 2015).

Considering the current local context, much of the public discussion in the public on migration trends and social changes takes place in regard to socio-political subjects concerning place, identity, power, and legal institutions (Mathews, 2020; South China Morning Post, 2021). At the same time, the study of Lui et al. (2022) also showed that socio-political variables like people’s social discontentment and perceived political efficacy could influence the mobility strategies of families, and thus their emigration intentions. To determine whether or not factors regarding these subjects could be as influential as they are portrayed as being, this study seeks to investigate how five socio-political factors—mobility, sense of place, inequality, trust and confidence in the law and the legal system, and sense of global citizenship—may act as the motivations promoting people’s intention to migrate. To systematically demonstrate the influence of these socio-political variables, we grouped them into three categories. The first category concerns the personal capacity of individuals, which is represented by their mobility in terms of cross-border and trans-border experiences; the second category concerns the personal orientations of individuals, which includes the variables of sense of place and sense of global citizenship; and the third category concerns perceptions of external environment, which is reflected by individuals’ trust and confidence in the law and the legal system, and their perception of inequality. In addition, identifying these drivers of migration from the perspective of a migration origin may ultimately help shed light on this surge in the outward movement of the population—a potential wave of emigration.

## Literature review

### Proposed motivations for migration and migration intention

#### Mobility

Migration has been widely studied in regard to its relationship with mobility and as a form of mobility (Bui & Wilkins, 2016; Gustafson, 2009; Lassen, 2006; Nagatomo, 2009). Mobility can take multiple forms, such as residential mobility, transnational mobility, and travel mobility. In particular, mobility in terms of international and outbound travel has been suggested to influence individuals’ intention to migrate (Gustafson, 2009). International or cross-border mobility is said to cause changes in one’s attitudes, orientations, and identities, as people may acquire different experiences, knowledge, and social interactions through traveling (Gustafson, 2009; Lassen, 2006; Mazzoni et al., 2018). These experiences and changes, in turn, may motivate people to pursue an alternative lifestyle and improved quality of life in another place, and thus trigger the intention of people to migrate (Bui & Wilkins, 2016). For example, Bui and Wilkins (2016) studied the migration pattern of young people in Japan and showed that repeated travel to a destination could motivate people’s intention to migrate. Nagatomo (2009) also suggested that Japanese individuals who experienced Australian lifestyles and culture during their trips could eventually be motivated to migrate to Australia as a result of their travel experience.

At the same time, cross-border mobility nowadays has become a diverse activity that people undertake for various purposes, including relaxation, vacations, business, employment, and studying (Frändberg, 2014; Nagatomo, 2009; Ono, 2009). Experiences from traveling for various purposes may influence people’s intention to migrate in different ways. Frändberg (2014) described how people with different purposes and experiences in regard to travel, in terms of employment, studying, and business, vary in their mobility and, subsequently, their chances of repeated travel and staying abroad. A recent study on the migration motives of Hong Kong residents to Greater Bay Area (GBA) cities showed that socio-political factors, such as individuals’ attitudes toward GBA cities and their prior mobility to and experiences with the region, could effectively predict their migration intention (Zhu et al, 2021).

#### Sense of place

Sense of place, also known as place attachment, describes the connection of people to a place which they experience and perceive to be meaningful (Jorgensen & Stedman, 2001; Kyle et al., 2005). Scholars have commonly conceptualized the attachment of people to a place as consisting of a few dimensions, including place identity, which describes individuals’ expression and affirmation related to identity; place affect, which reflects the emotional connection between a place and individuals; place dependence, which represents the functional attachment of individuals to a place; and place social bonding, which denotes individuals’ social bonding and social interactions with other individuals derived from a specific place (Jorgensen & Stedman, 2001; Kyle et al., 2005; Ramkissoon et al., 2013). In many cases, people with a stronger sense of place are less inspired to find a different place and way of living, and have lower incentives to migrate, as they find significant meaning or utility in their current place of residence (Petrović et al., 2017; Simms, 2017; Theodori & Theodori, 2014). Petrović et al. (2017) concluded in their research on citizens in eight cities in Serbia that people with lower levels of place attachment displayed a higher inclination to migrate.

While the positive association between sense of place and migration intention would hold in most cases, the four dimensions of sense of place may promote migration differently. Adams and Adger (2013) studied how benefits provided by the social environment of a place may determine people’s attachment to that place and, subsequently, their intention to migrate. The authors showed that less of a sense of place in terms of utility and commitment (similar to place dependence and place affect) would lead to a greater incentive to migrate. Similarly, Simms (2017) demonstrated in interviews with residents of Terrebonne Parish in Louisiana, USA, that people in the area showed a strong will to remain in their homeland, as they were highly attached to their place of residence in terms of their local culture and sense of identity. Theodori and Theodori (2014) also indicated that young people with strong attachments to their community, friends, and family generally show a higher tendency to stay than to migrate. On the other hand, in certain cases, a strong sense of place may not deter migration, due to other even stronger migration incentives. For instance, Eacott and Sonn (2006) found that rural Australians who perceive a sense of attachment to their community would still be motivated to migrate to urban areas to look for better work and education opportunities.

#### Inequality

Inequality is one of the most frequently discussed determinants of migration in the existing literature, especially in terms of income inequality (Black et al., 2005; Liebig & Sousa‐Poza, 2004; Stark, 2006). Other forms of inequality (e.g., gender inequality, education inequality, and racial inequality) have also been suggested to drive migration and individuals’ intention to migrate (Crowder & South, 2005; Williams, 2009). For example, Stark (2006) found that the incentives for people within a population to migrate were greater with a higher level of income inequality, as measured by the Gini coefficient. Among studies adopting inequality as a predictor, most employed some form of indices or national statistics and figures to quantify inequality; for example, income inequality is often represented by the Gini coefficient (Stark, 2006). While these indices provide an objective measurement, they do not take into consideration how individuals intending to migrate perceive and are affected by inequality. Assessing migration only on the macro and structural levels may fail to address individuals as active agents of migration who make conscious decisions (De Haas, 2011). That is, whether or not individuals know about and understand inequality in their place of residence and whether or not they perceive inequality as a problem could also be the keys to determining their intentions or decisions to migrate. These areas are rarely addressed by scholars.

Among one of the few exceptions, Cai and Wang (2008) assessed the influence of perceptions of inequality on migration in China through a case of rural to urban migration. The authors conceptualized inequality as migrants’ “individual migration motivation”. Inequality was measured by a few dimensions, including perceptions of exploitation at work, discrimination, the fairness of society, wage inequality, and social inclusion. Results of the study indicated that people who feel discriminated against and as though they are treated unequally have a higher tendency to migrate from rural to urban areas.

#### Trust and confidence in the law and the legal system

There is an established body of research on people’s trust and confidence in the law, legal authorities, and the legal system, spanning the areas of, for example, institutional justice, criminal justice, and the media (Tyler, 2001; Voicu & Tufiş, 2017; Wu, 2014). Yet, these studies have generally focused on gauging the level of trust and confidence of people in legal institutions from specific demographic backgrounds, such as ethnic minorities and new immigrants, and the factors that determine the different levels of trust and confidence (Tyler, 2001; Voicu & Tufiş, 2017; Wu, 2014). For instance, Tyler (2001) studied the institutional legitimacy of legal authorities, including the courts and the police, arguing that people tend to base their evaluations on their personal experiences with those authorities, including procedural fairness, and an institution’s ability to recognize people’s rights and treat people with dignity. Nevertheless, previous studies have yet to investigate whether or not and how public trust and confidence in the law and the legal system play a role as a predictor of people’s intention to emigrate.

The enactment of the National Security Law in July 2020 has drawn huge public attention to its effects on Hong Kong’s rule of law. With the law’s broad scope, uncertainties arose over its applicability to hitherto legal activities, and how various legal authorities, including the police, the courts, and the newly established Office for Safeguarding National Security, would operate under the new legal landscape (Hong Kong Free Press, 2021; South China Morning Post, 2021). Whether or not these drastic legal and political developments have strengthened or weakened Hong Kong people’s trust in legal authorities and, by extension, their desire to emigrate, is a question worthy of consideration.

#### Global citizenship

Global citizenship espouses the identities, practices, and responsibilities of people as world citizens who should understand and get involved in communities around the world in every respect (Davies, 2006; Oxfam n.d.; Oxley & Morris, 2013). Although literature explicitly ascertaining the associations between global citizenship involvements and identity and migration intentions is lacking, clues can be drawn from a similar case of transnational citizenship, the European Union (EU) citizenship. It was suggested in the case of EU citizenship that through social communications, involvement, and interactions with people of different nationalities and background, networks between people are created, thus a shared identity and sense of belonging can be formed in the community that is beyond local or national level (Mazzoni et al., 2018). In terms of empirical findings, correlations between EU participation and identification in terms of politics and economy, and people’s short- and long-term movements across EU countries were identified by Mazzoni et al. (2018). A similar study by Siklodi (2015) also showed that citizens recognizing a civic EU identity tend to be more active and mobile, while citizens who hold a national identity were more passive and likely to stay in the home country.

Under the context of the current study, we suggest that engagement in global citizenship issues may provide Hong Kong residents experiences in global involvement, and the development of an identity to be a global citizen. This global identity, deterritorialized in nature, provides an alternative to the identities at the local and national levels, such as ‘being a Hong Konger’ and ‘being a Chinese’, which citizens have to constantly juggle and which have been sources of disputes and conflicts in recent years (Chow et al., 2020). Therefore, with reference to the findings of previous literature, we propose that people’s practice and identification with global citizenship promote migration intentions.

## Methods

### Questionnaire survey design

A telephone survey randomly sampling the local population was performed with assistance from the Hong Kong Public Opinion Research Institute (PORI). The survey was conducted by the center using a web-based Computer Assisted Telephone Interview.^1^ The target population of the survey was Cantonese-speaking Hong Kong residents aged 18 years or above. All telephone interviews were carried out during the period from 7 May 2021 to 17 June 2021. A total of 801 completed responses were collected, consisting of 401 from landline numbers and 400 from mobile numbers. An effective response rate of 61.9% was yielded.

The survey was divided into three parts. In the first part, the key socio-political variables were investigated, including items on sense of place (A1–A16), inequality (B1–B4), trust and confidence in the law and the legal system (C1–C5), and global citizenship (D1–D6). To comprehensively represent the measured variables, items were designed to reflect multiple dimensions of the constructs. For questions related to inequality, items covered issues on income inequality, the gap between the rich and the poor, upward mobility, and social connections; for questions related to trust and confidence in the law and the legal system (C1–C5), items concerning the rule of law, legal procedures, and law enforcement units were included; and for questions related to global citizenship, items were designed to consider global issues associated with international news, ethnic minorities, charity, the environment, membership, and social movements. The variable of sense of place was sub-divided into four commonly employed sub-variables: place identity (A1–A3), place affect (A4–A7), place dependence (A8–A11), and place social bonding (A12–A15). References were drawn from past literature on the above constructs (Gustafson, 2009; Jorgensen & Stedman, 2001; Mazzoni et al., 2018; Tyler & Jackson, 2014), with amendments made according to the local context. All of these items were measured on a five-point Likert scale, where a score of one represents the lowest level of agreement and a score of five represents the highest level of agreement.

The second part consists of items concerning the mobility and migration intention of respondents. The mobility of respondents was measured in terms of cross-border mobility, in which their frequency of traveling in the past to mainland China and other destinations was explored (E1 & E2). Similarly, the intention of respondents to migrate to two destination choices—mainland China and destinations other than mainland China—was measured (F1 & F2). With a close connection to Hong Kong both geographically and culturally, mainland China has long been one of the destinations that Hong Kong residents visit the most (Census & Statistics Department, 2003), as well as a popular place of residence. Therefore, the mobility and migration intention of respondents to mainland China were measured separately from destinations in the rest of the world. These items were measured on a five-point Likert scale, in terms of frequency and duration of mobility, and in terms of level of agreement in migration intention. The last part of the questionnaire collected the demographic information of respondents, including their age, level of education, and monthly household income.

### Data analysis

Stepwise multiple regression analysis consisting of three models was carried out. The first regression model included only the variable regarding personal capability (i.e., mobility). Subsequently, in the second and third models, factors regarding personal orientations (i.e., sense of place and global citizenship) and perceptions of the external environment (i.e., inequality and trust and confidence in the law and the legal system) were entered into the regression analysis consecutively. To confirm the reliability of the questionnaire items, the Cronbach’s alpha values of all constructs with multiple items were computed. All statistical tests were performed with SPSS 26.0.

## Results

### Demographic characteristics of respondents

According to Table 1, female respondents (57.6%) in this questionnaire slightly outnumber that of males (42.4%). In terms of age, respondents in age groups below 60 years were mostly distributed equally, but a larger proportion of respondents aged 60 or above was observed (37.5%). Regarding education levels, the majority of the respondents had attained certain forms of post-secondary education (42.9%). A fairly larger number of respondents had a monthly household income of HK$1–9999 (24%) and HK$10,000–19,999 (18.6%).

**Table 1:** Demographic characteristics of participants

Gender	N	%	Education level	N	%
Male	340	42.4	Primary or below	90	11.2
Female	461	57.6	Lower secondary	119	14.9
			Upper secondary	233	29.1
			Post-secondary: Non-degree	77	9.6
			Post-secondary: Degree	267	33.3
			Refuse to answer	15	1.9

Age groups			Household income*		
18–19	25	3.1	No income	99	12.4
20–29	120	15	HK$1–9,999	192	24
30–39	109	13.6	HK$10,000–19,999	149	18.6
40–49	108	13.5	HK$20,000–29,999	97	12.1
50–59	109	13.6	HK$30,000–49,999	100	12.5
60 or above	300	37.5	HK$50,000–99,999	50	6.2
Refused to answer	30	3.7	HK$100,000 or above	20	2.5
			Refused to answer	94	11.7
			Total	801	100

*Officially pegged exchange rate: US$1.00 = HK$7.80

### Socio-political determinants of intention to migrate

Among the sub-constructs of sense of place, respondents showed a particularly high level of place social bonding (mean score = 4.03), while place identity, place affect, and place dependence had similar mean scores of around 3.5 (Table 2). Respondents generally perceived high levels of inequality, with a mean score of 3.73. Items concerning income inequality (B1 & B2) also had particularly high scores. A moderate level of trust and confidence in the law and the legal system was identified (mean score = 2.97). Meanwhile, a relatively low level of sense and practice of global citizenship was reported by respondents (mean score = 2.06). Respondents did engage in global citizenship practices more often in terms of following global news and affairs and in regard to environmental issues. The Cronbach’s alpha coefficients for all constructs except inequality exceeded the commonly referenced threshold of 0.6 (Taber, 2018). The alpha coefficient of the inequality construct was 0.582. However, considering that the construct and items were newly developed, with adaptations to a local context, this level of reliability should still be acceptable.

**Table 2:** Distribution of responses in questionnaire items

Item	Strongly agree	Agree	Neutral	Disagree	Strongly disagree	Mean score	Cronbach’s alpha
*Place identity*						3.41	0.65
A1. All the things in Hong Kong represent me	14	17.3	21.3	26	21.5	2.76	
A2. I can truly be myself when I am in Hong Kong	31.2	28.3	14.3	14.1	12.1	3.52	
A3. I am proud of being a Hong Konger	43.2	27.5	15.2	8.4	5.6	3.94	
*Place affect*						3.54	0.718
A4. I want to stay in Hong Kong instead of moving to other places	40.3	25.2	13.9	10.6	10	3.75	
A5. I enjoy living in Hong Kong	35.4	29.8	16.1	11	7.7	3.74	
A6. There are other places that are more suitable for living than Hong Kong. (R)	33.6	29.7	10.6	12.6	13.5	2.43	
A7. I miss Hong Kong when I am away	51.3	32	9	4.3	3.4	4.23	
*Place dependence*						3.35	0.603
A8. My daily needs can be satisfied in Hong Kong	46.8	33.7	10.9	5.7	2.9	4.16	
A9. Hong Kong allows me to do things that I cannot do in other places	23.3	27.3	16.1	19.8	13.4	3.27	
A10. I cannot do some of the things that I want to do when staying in Hong Kong. (R)	31.1	35.2	12.1	10.7	10.9	2.35	
A11. Hong Kong provides different opportunities for me	29.2	32.9	17.3	11.4	9.2	3.61	
*Place social bonding*						4.03	0.641
A12. The social networks I developed in Hong Kong are important to me	49.1	34.2	9	5.8	1.9	4.23	
A13. People who I want to stay in contact with are in Hong Kong	41.6	29.9	12.4	11.3	4.8	3.92	
A14. In Hong Kong, I can easily find people who share my interests	36	37.1	14.1	8.9	3.9	3.92	
A15. People who are important to me are staying in Hong Kong	46.3	30.6	9.1	8.9	5.1	4.04	
*Inequality*						3.73	0.582
B1. Differences in income in Hong Kong are too large	53.7	33.8	6	4	2.5	4.32	
B2. Disparity between the rich and the poor in Hong Kong has grown in the past five years	55.2	31.9	5.3	4.6	3	4.32	
B3. There are enough opportunities for upward mobility in Hong Kong for people to become successful	11.3	27.2	22.1	21.7	17.6	3.07	
B4. Hard work doesn’t generally bring success; it’s more a matter of luck and connections	18.4	29.8	20	18.4	13.4	3.22	
*Trust and confidence in the law and the legal system*						2.97	0.905
C1. The law protects the rights of people in power rather than those of ordinary citizens. (R)	24.7	23.4	17	17.7	17.3	2.80	
C2. People in power use the law to try to control ordinary citizens. (R)	29.2	21.8	13.7	15.9	19.4	2.74	
C3. The law protects the interests of all citizens	20.4	22.3	19.1	17.7	20.4	3.05	
C4. The law is made through a fair procedure	22.2	26.8	16.8	13.1	21.1	3.16	
C5. Judges generally deliver reasonable judgements	15.4	28.7	21.4	17.6	16.8	3.08	

	Always	Often	Sometimes	Seldom	Never		
*Global citizenship*						2.06	0.621
D1. I keep up with global affairs by reading international news	15.1	44.4	23.7	7.3	9.5	3.48	
D2. I donate to international non-governmental organizations with global aims	1.9	5.9	16.2	17.6	58.4	1.75	
D3. I participate in activities that enhance the understanding and welfare of ethnic minorities	0.6	1.8	9.5	21.3	66.7	1.48	
D4. I do things (e.g., recycling at home or buying second-hand products) for environmental purposes	5.9	27.7	36	14.8	15.7	2.93	
D5. I am a member of a non-governmental organization with global aims	0.1	0.5	2.5	5.9	91	1.13	
D6. I use the internet or social media to participate in global social movements (e.g., related to poverty, human rights, democracy, and anti-human trafficking, etc.)	0.4	4.3	13.8	14.5	67	1.57	
*Mobility*							
E1. How often did you visit mainland China before the social movements in 2019 and COVID-19 occurred?	4.9	18.3	29.2	30.2	17.4	2.63	
E2. How often did you visit destinations other than mainland China before the social movements in 2019 and COVID-19 occurred?	2	17.9	34.9	21.3	23.9	2.53	

*Migration intention*	Very much agree	Somewhat agree	Neither agree nor disagree	Somewhat disagree	Very much disagree		
F1. I intend to migrate to mainland China within the next two years	4	5.2	6.4	19.2	65.3		
F2. I intend to migrate internationally to destinations other than mainland China within the next two years	8.4	9.7	12.5	21.4	48		

*R:* reverse coded

The travel mobility of respondents in terms of the frequency with which they traveled to mainland China and other destinations were similar; respondents who traveled “sometimes” and “seldom” outside Hong Kong were the majority, and around 20% of respondents never traveled.

Around 20% of respondents showed stronger intentions to migrate internationally to destinations other than mainland China, and approximately 10% showed interest in migrating to mainland China. Around 65% and 50% of respondents did not have any plan to move to mainland China or other destinations, respectively, in the coming years. Possible associations between age and intention to migrate internationally and to mainland China were observed, where a greater number of older respondents intend to migrate to mainland China, and more younger respondents intend to migrate internationally.

### Stepwise multiple regression analysis: Intention to migrate to mainland China

In the first model (Table 3), the personal capability of respondents in terms of mobility was tested against their intention to migrate to mainland China. A positive relationship was identified between respondents’ frequency of traveling to mainland China and their intention to migrate there (*p* < 0.01). In particular, mobility experiences with the purposes of entertainment (*p* < 0.05) and tours and vacations (*p* < 0.01) promoted people’s intention to move to mainland China. In the second model, the personal orientations of individuals in terms of sense of place and global citizenship were entered into the regression analysis. Place dependence (*p* < 0.05) and place social bonding (*p* < 0.05), showed significant positive and negative associations, respectively, with people’s intention to move to mainland China. People’s sense of global citizenship was negatively associated with their intention to migrate to mainland China (*p* < 0.05). The effects of the mobility items on visitation frequency remained, while those of the item on entertainment travel experience was absorbed by the additional variables. In the full model, all variables were included and only four paths showed significant results, including mobility in terms of visitation frequency to mainland China (*p* < 0.05), global citizenship (*p* < 0.05), trust and confidence in the law and the legal system (*p* < 0.01), and inequality (*p* < 0.01). Considering the R^2^ value for the three models, the personal capacity of people in terms of mobility contributed the most to the total variance (13.6%). The other two groups—personal orientation (4.1%) and perceptions of the external environment (3.5%)—showed similar levels of effects.

**Table 3:** Stepwise regression analysis between socio-political variables and individuals’ intention to migrate to mainland China

	Model 1 (personal capacity)	Model 2 (personal capacity + personal orientation)	Model 3 (personal capacity + personal orientation + perception of external environment)
Mobility experience (mainland China)			
Visitation frequency	0.157**	0.130*	0.164*
Work	− 0.026	− 0.018	− 0.036
Study	0.059	0.075	0.050
Visiting friends and family	0.078	0.067	0.059
Entertainment	0.101*	0.084	0.072
Tours and vacations	0.162**	0.121*	0.077
Mobility experience (destinations other than mainland China)			
Visitation frequency	-0.087	− 0.071	− 0.047
Work	0.084	0.086	0.089
Study	− 0.056	− 0.040	− 0.016
Visiting friends and family	− 0.065	− 0.061	− 0.081
Entertainment	0.028	0.049	0.058
Tours and vacations	− 0.048	− 0.020	− 0.031
Sense of place			
Place identity		0.078	0.045
Place affect		0.041	− 0.001
Place dependence		0.139*	0.039
Place social bonding		− 0.107*	− 0.096
Global citizenship		− 0.110*	− 0.091*
Trust and confidence in the law and the legal system			0.155**
Inequality			− 0.151**
F statistics	6.572	6.614	7.380
R	0.400	0.456	0.496
Adjusted R^2^	0.136	0.177	0.212

***p < 0.001, **p < 0.01, *p < 0.05

### Stepwise multiple regression analysis: Intention to migrate internationally to destinations other than mainland China

Model 1 in Table 4 shows that the visitation frequency of people to mainland China negatively influenced people’s intention to migrate internationally to other destinations (*p* < 0.05). Visitation frequency to other destinations, however, was positively associated with their intention to migrate internationally (*p* < 0.01). In particular, mobility experience in regard to work (*p* < 0.05) and study (*p* < 0.05) in other destinations promoted people’s intention to migrate internationally, while greater mobility experience related to tours and vacations (*p* < 0.05) and visiting friends and family (*p* < 0.05) in mainland China discouraged international migration. In the second model, place identity (*p* < 0.01) and place affect (*p* < 0.001) showed negative associations with people’s intention to migrate. Sense of global citizenship was positively associated with migration intention (*p* < 0.01). In the final model, the added variable of trust and confidence in the law and the legal system negatively predicted people’s intention to migrate (*p* < 0.01). In this model, place affect (*p* < 0.001), global citizenship (*p* < 0.05), and visitation frequency to other destinations (*p* < 0.01) also showed significant results. The effects of the variables on mobility experiences with particular purposes, visitation frequency to mainland China, and place dependence were absorbed by the additional factors in Models 2 and 3. The total variance explained for the three models was 17.3%, 42.8%, and 44.3%, respectively.

**Table 4:** Stepwise regression analysis between socio-political variables and individuals’ intention to migrate internationally

	Model 1 (personal capacity)	Model 2 (personal capacity + personal orientation)	Model 3 (personal capacity + personal orientation + perception of external environment)
Mobility experience (mainland China)			
Visitation frequency	− 0.123*	− 0.057	− 0.077
Work	0.025	0.008	0.019
Study	0.039	− 0.005	0.009
Visiting friends and family	− 0.112*	− 0.082*	− 0.073
Entertainment	− 0.030	− 0.010	− 0.008
Tours and vacations	− 0.156	− 0.030	0.000
Mobility experience (destinations other than mainland China)			
Visitation frequency	0.187**	0.159*	0.141**
Work	0.113*	0.048	0.045
Study	0.118*	0.096*	0.083*
Visiting friends and family	0.020	0.005	0.020
Entertainment	0.001	− 0.040	− 0.041
Tours and vacations	0.027	− 0.031	− 0.024
Sense of place			
Place identity		− 0.006	0.016
Place affect		− 0.446***	− 0.414***
Place dependence		− 0.142**	− 0.075
Place social bonding		0.031	0.025
Global citizenship		0.102**	0.089*
Trust and confidence in the law and the legal system			− 0.154**
Inequality			0.036
F statistics	8.290	20.571	19.741
R	0.440	0.671	0.682
Adjusted R^2^	0.170	0.428	0.442

***p < 0.001, **p < 0.01, *p < 0.05

## Discussion and conclusion

This study employed five socio-political variables to predict the migration intention of Hong Kong people under the suggested new wave of migration. The results generally indicated that determinants in the three proposed aspects, from personal capability to personal orientation and perceptions of the external environment, all played significant roles in shaping people’s intention to migrate. These findings also confirm that the recent socio-political changes in Hong Kong could be major drivers of this potential wave of migration.

In terms of respondents’ mobility experiences, a higher frequency of visiting a destination was found to promote people’s intention to migrate to that destination, which is consistent with findings from past literature (Bui & Wilkins, 2016). Repeated visits may already indicate some form of attachment to the destination (Isa et al., 2019), which could in turn trigger people’s intention to migrate. While the visitation frequency of individuals positively predicted their intention to migrate to the respective destinations, the purposes of past trips showed that people intending to move to the two destinations under study—mainland China and overseas—may look for different lifestyles and potential areas of development. Respondents more motivated to migrate internationally reported higher levels of mobility experience in terms of studying and work. This may suggest that future career and personal development could be the primary purpose of migration for this group of respondents intending to migrate internationally, as their past experience has led them to recognize different opportunities and alternative lifestyles available in their migration destination. On the other hand, individuals with greater intention to move to mainland China generally had more experience of entertainment, tours, and vacations in their past visits to the mainland. Recognizing the recreational opportunities in mainland China, migration for this group is more leisure-oriented, potentially as a change of lifestyle upon retirement. Mainland China has long been a destination for people looking to settle following retirement, and it is no surprise that its leisure and recreational environment is motivation for migration for people in Hong Kong. This may also explain the lower level of intention of people with more international travel experiences to migrate to mainland China, due to the vastly different opportunities and lifestyles available among the destinations.

The positive association between place dependence and intention to move to mainland China may imply that, for these individuals, mainland China could be deemed a similar but improved version of Hong Kong in terms of its living environment and cheaper living costs. Instead of aiming for completely different lifestyles, this group of individuals may look for a better quality of life materialistically and culturally in mainland China. In the same sense, international migration that likely involves more drastic lifestyle and social adjustments was discouraged by higher levels of place dependence. With closer connections to friends and family in Hong Kong reflected by higher levels of place social bonding, people were less motivated to move to mainland China. This result is very much in line with existing studies on sense of place, which showed that attachment to the local community, friends, and family could deter rural to urban or international migration (Simms, 2017; Theodori & Theodori, 2014). However, considering the geographical proximity, well-established transport network, and relaxed border control between mainland China and Hong Kong under the concept of the Guangdong-Hong Kong-Macau Greater Bay Area, it is unexpected to find social connections being a major concern discouraging people’s migration intention. People may still draw a relatively clear distinction between Hong Kong and mainland China geographically, and frequent traveling between the two places following migration has yet to be an option considered by most.

Trust and confidence of people in the law and the legal system influenced people’s migration intention to the two destination choices in opposing direction, where higher levels of trust and confidence are correlated with a stronger tendency to migrate to mainland China, while lower levels of trust and confidence drove people to consider international migration. These results indicated that people’s concerns about recent political changes and the wellbeing of the legal system could be one of the reasons driving international migration, which has been suggested by overseas observers and researchers (Bloomberg, 2021; Lui et al., 2022). Hong Kong’s sound legal system has been a symbol of its promised “high degree of autonomy” under the “One Country, Two Systems” and a foundation for its reputation as a world-class financial hub. Diminishing public trust and confidence in the legal system may also mean rising concerns about Hong Kong’s declining autonomy and a gradual loss of advantages and opportunities in international finance and business. Moreover, distrust in legal and political authorities has been widely suggested to lead to a lower level of compliance with the law among the general public (Lee & Lo, 2020; Tyler, 2001). Under the new era of national security, some kinds of behavior—for example, the publication of “seditious” children’s books (The Guardian, 2021)—may result in previously unthought of criminal liabilities, and may therefore prompt some individuals to consider migration. On the other hand, a higher degree of trust and confidence in the legal system appeared to increase people’s intention to move to mainland China. This high level of trust and confidence may largely originate from the recent legal and political reforms in Hong Kong following the enactment of the National Security Law, which is Chinese national legislation by nature. Under this new legal regime, the Hong Kong SAR Government has greater control over the judicial process concerning infringements of national security offences (e.g., the Chief Executive’s power to appoint a panel of judges to precede over cases such as these), which is said to be helping to rebuild Hong Kong’s stability after widespread protests for the better part of 2019 (The Government of the Hong Kong Special Administrative Region, 2021). The fact that this group of individuals still intends to move to mainland China may indicate that people are more confident about the governing ability of the Chinese government than the Hong Kong SAR government, among other things. The real challenge for the latter is that, no matter whether people in Hong Kong show a higher or lower degree of trust in the law and the legal authorities, a considerable number of them still intend to emigrate, whether to mainland China or elsewhere. In fact, result in this part could be relevant to the latest policy address of Hong Kong, where the government outlined an array of strategies and initiative to attract talents from outside and enhance the city’s competitiveness, in response to the increasing movement of population (The Government of the Hong Kong Special Administrative Region, 2022). Yet, our results have shown that retaining talents originally in Hong Kong could be a pressing, if not more urgent, issue than drawing talents internationally to Hong Kong.

Findings on the relationship between global citizenship and people’s intention to migrate could reflect the way in which the values that people hold could be associated with their potential destination choice in their migration. A stronger sense of global citizenship may reflect the way in which people hold more liberal and Western-oriented values, while the opposite could signify values that are relatively more nationalistic and conservative. While societies more prevalently embracing certain sets of values could be choices for respective potential migrants, a dilemma again appears; in the eyes of some individuals, Hong Kong is not “global” enough, while, from the perspectives of others, Hong Kong is too “global”. Having that said, nationalism and multiculturalism might not necessarily be opposing concepts, as a person with a stronger local and national identity may still have a strong sense of global citizenship (I. Davies et al., 2018). However, as a world-known international city in previous decades, Hong Kong might gradually be failing to provide an environment that accommodates people with diverse values and cultures and that allows local, national, and global identities to be formed simultaneously.

Unlike other determinants, the perception of inequality only influenced people’s intention to move to mainland China, not internationally. A stronger concern about inequality in Hong Kong is associated with a lower intention to migrate to mainland China. This finding can be explained by the way in which many people hold the politicized belief that the biased economic structure in Hong Kong is a consequence of economic integration with mainland China (e.g., Wong et al., 2021). Conversely, two reasons can explain the effect of the lack of inequality on migration to other countries. First, it might simply be the case that people do not migrate to certain destinations because of political reasons, instead of inequality. Second, while migration has been one of the solutions for people looking to escape unequal social or economic environments, migrants have continuously been reported to suffer further from unequal treatments and policies in their migration destination (Gross-Wyrtzen, 2020; Pham et al., 2019). That being said, for individuals who are concerned about inequality, migration may not always be a solution and may even be a worse choice than staying.

The current study primarily focused on discussing Hong Kong as the origin of migration and has investigated only two destination choices of migration: mainland China and other international destinations. While comparisons between migration intentions in international migration and migration to mainland China can help reflect the differences among individuals, as shown by the findings of this study, one limitation is that differences most certainly also exist between various “international” destinations, which cannot be easily generalized. To more thoroughly understand how migration origins and destinations may interact in shaping migration, future studies may consider sampling the destination choices of respondents by exact location or by region, which could then allow for a more detailed analysis. At the same time, the current study focused on investigating the relationships between migration intention and the five socio-political determinants respectively, yet, the interactions between determinants were not explored. To better acknowledge the complexity in the relationships between the determinants and migration intentions, future studies may take a further step to analyze the interactions between any adopted determinants.

To better understand the recent surge of emigration in Hong Kong, the current study adopted a multi-level framework with five determinants that correspond to the current socio-political context of Hong Kong. Stepwise multiple regression was carried out and the results derived once again demonstrated the complexity in people’s migration intentions. Socio-political factors regarding people’s personal capacity, personal orientations, and perceptions of external environment all contributed to explaining people’s intention to migrate, showing that socio-political shifts in Hong Kong could be one of the current drivers of migration. Both high and low levels of perceptions concerning a few variables, including mobility, trust and confidence in the law and the legal system, and sense of global citizenship, were shown to influence people’s intention to migrate to either of the migration destinations under study, which in turn indicates that the ultimate challenge for Hong Kong is addressing the increasing tendency of people to migrate with diverse motivations. The current study also contributed to the body of migration research by showcasing the influence of socio-political factors in migration with the unique case of Hong Kong.

## Data Availability

The datasets used and/or analysed during the current study are available from the corresponding author on reasonable request.

## Footnotes

1 To allow for random sampling and reduce sampling bias, telephone numbers were randomly generated using known prefixes assigned to telecommunication services providers under the Numbering Plan provided by the Office of the Communications Authority (OFCA). Both landline and mobile numbers were included in the sampling frame, with a target ratio of one to one. If more than one eligible individual was available in the household of a landline sample, the “next birthday rule” was applied, where the person with a birthday closest to the survey date was interviewed.
